# Risk Factors and VEGF, hs-CRP, and ESR in Central Serous Chorioretinopathy

**DOI:** 10.1155/2024/9322594

**Published:** 2024-09-19

**Authors:** Lingjuan Sun, Li Yin, Shurui Wang, Haiyan Wang

**Affiliations:** Department of Ophthalmology Shijiazhuang People's Hospital, Shijiazhuang, Hebei 050000, China

## Abstract

**Objective:**

This study aimed to investigate the risk factors associated with central serous chorioretinopathy (CSC) and analyze the relationship between vascular endothelial growth factor (VEGF), high-sensitivity C-reactive protein (hs-CRP), erythrocyte sedimentation rate (ESR), and CSC.

**Methods:**

A total of 109 patients diagnosed with CSC (CSC group) at our ophthalmology clinic from February 2017 to February 2021 were included, with 103 volunteers from our hospital's health examination center serving as the control group. Additionally, the new multimodal imaging classification of 109 CSC patients was further divided into simple CSC (57 cases) and complex CSC (52 cases). Demographic data, underlying diseases, medical history, and medication history were collected. Levels of VEGF, hs-CRP, and ESR were measured, and multifactorial logistic regression analysis was performed to identify factors influencing CSC. Receiver operating characteristic (ROC) curve analysis was conducted to assess the diagnostic value of VEGF, hs-CRP, and ESR in CSC.

**Results:**

The CSC group showed a higher proportion of males, smoking history, alcohol consumption, comorbid obstructive sleep apnea, hypothyroidism, renal disease, *Helicobacter pylori* infection, steroid use, and shift work compared to the control group (*P* < 0.05). VEGF, hs-CRP, and ESR levels were significantly higher in the CSC group than in the control group (*P* < 0.05). The levels of VEGF, hs-CRP, and ESR in the complex CSC group were higher than those in the simple CSC group (*P* < 0.05). Male gender, shift work, *Helicobacter pylori* infection, hypothyroidism, elevated VEGF, hs-CRP, and ESR were identified as risk factors for CSC (*P* < 0.05). The combined diagnostic value of VEGF, hs-CRP, and ESR (area under the ROC curve: 0.886) was higher than that of individual markers (0.722, 0.728, and 0.703, respectively) (*P* < 0.05).

**Conclusion:**

Male gender, shift work, *Helicobacter pylori* infection, hypothyroidism, and elevated levels of VEGF, hs-CRP, and ESR are risk factors for CSC. The combined use of VEGF, hs-CRP, and ESR demonstrates higher diagnostic efficiency in identifying CSC.

## 1. Introduction

Central serous chorioretinopathy (CSC) is a choroidal retinal disease characterized by the accumulation of subretinal fluid leading to localized serous detachment of the macula, with or without focal serous detachment of the retinal pigment epithelium [1, 2]. CSC primarily affects young men between the ages of 20 and 45, with a higher prevalence in those experiencing significant stress or using corticosteroids [3, 4]. Although CSC often resolves spontaneously within several months, its propensity for recurrence poses a risk of progressive visual impairment [5], necessitating a deeper understanding of its pathophysiology and risk factors.

The pathogenesis of CSC is complex and multifactorial. Studies suggest that increased choroidal vascular permeability plays a crucial role in the development of CSC [1, 6, 7]. This increased permeability can result from insufficient choroidal capillary perfusion, which triggers inflammatory responses and subsequent localized serous detachment in the macula, manifesting as CSC symptoms [8]. Choroidal thickening is typically accompanied by vascular dilation and increased permeability, which exert mechanical pressure on the retinal pigment epithelium (RPE). This pressure compromises the nutrient supply to the RPE, leading to impaired RPE cell function [9]. Therefore, choroidal thickening is an important contributing factor to the development of CSC [10]. The extent of retinal functional damage caused by CSC is typically assessed using indicators such as best-corrected visual acuity (BCVA), central macular thickness (CMT), subretinal fluid (SRF) thickness, and outer retinal layer thickness [11, 12]. Despite the self-limiting nature of acute CSC, recurrent episodes can cause permanent damage to the retina, underscoring the importance of identifying and managing risk factors effectively.

Vascular endothelial growth factor (VEGF) is known to be a significant mediator of vascular permeability and angiogenesis [13]. Elevated levels of VEGF have been associated with increased retinal vascular permeability, contributing to the pathogenesis of several retinal diseases, including CSC [14]. Similarly, high-sensitivity C-reactive protein (hs-CRP), a sensitive marker of systemic inflammation, has been implicated in various inflammatory and cardiovascular conditions [15, 16]. Elevated serum hs-CRP levels have been linked to the development and severity of diabetic retinopathy, suggesting a potential role in other retinal diseases such as CSC [17]. Furthermore, the erythrocyte sedimentation rate (ESR) is a nonspecific marker of inflammation that increases in response to acute and chronic inflammatory processes [18]. Elevated ESR levels have been associated with various inflammatory conditions [19], although its specific relationship with CSC remains to be fully elucidated.

This study aims to investigate the risk factors associated with CSC and to analyze the relationships between VEGF, hs-CRP, and ESR levels and the occurrence of CSC. By understanding these associations, we hope to elucidate the potential diagnostic value of these biomarkers in CSC. This could provide valuable insights for the clinical management and prevention of CSC, ultimately improving patient outcomes. Specifically, we aim to determine whether a combined diagnostic approach using VEGF, hs-CRP, and ESR can enhance the diagnostic accuracy for CSC compared to using these markers individually.

## 2. Materials and Methods

### 2.1. Study Design and Participants

This study was conducted at the Ophthalmology Department of Hebei Shijiazhuang People's Hospital. A total of 109 patients diagnosed with central serous chorioretinopathy (CSC) between February 2017 and February 2021 were enrolled in the CSC group. The disease duration was 8.98 months (range: 4.39–12.29 months). An additional 103 healthy volunteers who underwent routine health check-ups during the same period were recruited as the control group. All participants provided informed consent, and the study protocol was approved by the hospital's Ethics Committee.

Inclusion criteria for the CSC group were as follows:

  Presence of subretinal fluid (SRF) detected by optical coherence tomography (OCT), with or without pigment epithelial detachment (PED);
  Fluorescein fundus angiography (FFA) showing clear fluorescein leakage from the retinal pigment epithelium (RPE);
  Indocyanine green angiography (ICGA) indicating late-phase focal choroidal hyperpermeability;
  All CSC patients included in this study were treatment-naïve;
  Age 18 years or older;
  Written informed consent.

Exclusion criteria for both groups included:

  Presence of hemorrhage, age-related macular degeneration, or polypoidal choroidal vasculopathy;
  History of cataracts, glaucoma, diabetic retinopathy, or other significant ocular pathologies;
  Ocular surface inflammation, vitritis, or severe dry eye syndrome;
  History of malignancy, infectious diseases, or autoimmune disorders.
  Pregnant women.

### 2.2. Data Collection

Demographic data and medical histories, including smoking status, alcohol consumption, pregnancy status, underlying diseases (hypertension, diabetes, and hyperlipidemia), comorbid conditions (obstructive sleep apnea, hypothyroidism, kidney disease, and *Helicobacter pylori* infection), history of corticosteroid use, and shift work, were collected through structured interviews and medical record reviews.

### 2.3. Ocular Examinations

At the initial visit, all study participants underwent comprehensive ocular examinations, which included the following:

1. Optical Coherence Tomography (OCT): Spectral-domain OCT was performed using the Spectralis HRA + OCT device (Heidelberg Engineering, Germany) to measure the subfoveal choroidal thickness. Patients were instructed to fixate on the central fixation point while the lens was positioned close to the eye. Horizontal and vertical scans of the fovea were obtained using automated real-time eye-tracking technology. Choroidal thickness was measured from the outer border of the RPE hyperreflective line to the visible inner scleral surface. The software also analyzed the retinal microstructure, pigment epithelium/Bruch's membrane complex, and any presence of choroidal neovascularization.
2. Fluorescein Fundus Angiography (FFA): FFA was conducted using a fundus camera provided by TOPCON. Patients were premedicated with an oral antihistamine (chlorpheniramine) and administered compound tropicamide eye drops for pupil dilation. Baseline nonred fundus photographs were taken, followed by a slow intravenous injection of 1 : 2000 fluorescein sodium solution into the antecubital vein. If no allergic reactions or other adverse symptoms occurred, a 3 mL bolus of 20% fluorescein sodium was rapidly injected intravenously. The FFA procedure was initiated by pressing the timing button, capturing images to assess the type of leakage, lesion size, distribution, and any choroidal neovascularization [20].
3. Fundus Autofluorescence (FAF): FAF imaging was performed using the Optos imaging system (Optos, UK). After pupil dilation, patients were positioned in front of the device, aligning the eye with the imaging lens while focusing on the fixation target. The system captured images at multiple angles using specific excitation wavelengths to stimulate autofluorescence in retinal substances. The collected images were then uploaded to the computer system for evaluation by the clinician.

### 2.4. Laboratory Measurements

Venous blood samples (5 mL) were collected from all participants in the morning following overnight fasting. For serum VEGF and hs-CRP measurements, 3 mL of blood was allowed to clot at room temperature before centrifugation at 1200 × *g* for 5 minutes. Serum samples were stored at −80°C until analysis.

VEGF Measurement: Serum VEGF levels were measured using an enzyme-linked immunosorbent assay (ELISA) kit (Shanghai Enzyme-linked Biotechnology Co., Ltd.) according to the manufacturer's instructions. Standards, samples, and controls were added to microplate wells, followed by enzyme conjugate. The mixture was incubated at room temperature for 60 minutes. After washing, substrate solutions were added, and the reaction was stopped after 15 minutes. The absorbance was measured at 450 nm using a microplate reader (FK-SY96S, Fangke Instruments, Shandong, China), and VEGF concentrations were calculated based on a standard curve.

hs-CRP Measurement: High-sensitivity C-reactive protein levels were determined using a turbidimetric immunoassay on a Modular P800 analyzer (Roche Diagnostics, Switzerland). The reagent kit was provided by Beijing ReBio Science Co., Ltd.

ESR Measurement: Erythrocyte sedimentation rate was measured using the NF-9906 automated erythrocyte sedimentation analyzer (Chongqing NanFang Numerical Control Equipment Co., Ltd.). Blood samples were collected in EDTA tubes and analyzed according to the manufacturer's protocols.

### 2.5. Statistical Analysis

All statistical analyses were performed using SPSS version 25.0 (IBM Corp., Armonk, NY, USA). Continuous variables were tested for normality using the Kolmogorov-Smirnov test and expressed as mean ± standard deviation (SD). Comparisons between the CSC and control groups were made using independent sample *t*-tests or chi-square tests as appropriate. Multivariable logistic regression analysis was conducted to identify independent risk factors for CSC. Variables with a *P* value <0.05 in univariate analysis were included in the multivariable model. The diagnostic value of VEGF, hs-CRP, and ESR for CSC was assessed using receiver operating characteristic (ROC) curve analysis. The area under the ROC curve (AUC) was calculated to evaluate the performance of each marker individually and in combination. Delong test in MedCalc 20.0 software was used to compare the AUC differences between models. A *P* value <0.05 was considered statistically significant.

## 3. Results

### 3.1. Comparison of Baseline Characteristics between CSC and Control Groups

The proportion of gender, individuals with a history of smoking and alcohol consumption, and those with comorbidities such as obstructive sleep apnea, hypothyroidism, kidney disease, and *Helicobacter pylori* infection was significantly higher in the CSC group compared to the control group (*P* < 0.05). However, there were no statistically significant differences in age, pregnancy status, underlying diseases, or antidepressant use between the two groups (*P* > 0.05). As shown in Table 1.

### 3.2. Comparison of VEGF, hs-CRP, and ESR Levels between CSC and Control Groups

**Figure 1:**
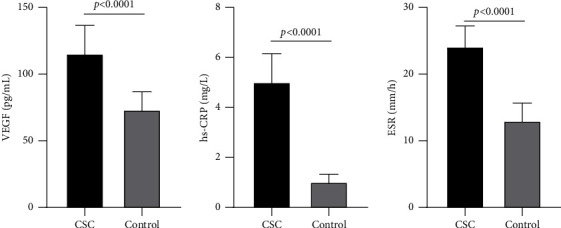
Differences in VEGF, hs-CRP, and ESR between CSC group and control group (mean ± sd). Note: central serous chorioretinopathy (CSC).

The levels of VEGF, hs-CRP, and ESR were significantly elevated in the CSC group compared to the control group (*P* < 0.05). Specifically, the mean VEGF level was 115.42 ± 21.09 pg/mL in the CSC group, whereas it was 73.26 ± 13.54 pg/mL in the control group. Similarly, the mean hs-CRP level was 5.02 ± 1.13 mg/L in the CSC group, significantly higher than the control group's mean of 1.02 ± 0.31 mg/L. Additionally, the mean ESR level was 24.15 ± 3.09 mm/h in the CSC group, whereas it was 13.02 ± 2.65 mm/h in the control group, as shown in Figure 1. These findings suggest that elevated levels of VEGF, hs-CRP, and ESR may be important factors in the pathogenesis of CSC.

### 3.3. Comparison of Baseline Characteristics between Simple CSC and Complex CSC Groups

Subsequently, the CSC group was further divided into the simple CSC group (*n* = 57) and the complex CSC group (*n* = 52) according to the new multimodal imaging classification. There were no statistically significant differences between the two groups in terms of gender, smoking history, alcohol consumption history, presence of obstructive sleep apnea, hypothyroidism, kidney disease, *Helicobacter pylori* infection, history of corticosteroid use, night shift work, age, pregnancy, underlying conditions, or use of antidepressants (*P* > 0.05), as shown in Table 2. Additionally, it was found that the levels of VEGF, hs-CRP, and ESR were significantly higher in the complex CSC group compared to the simple CSC group (*P* < 0.05), as shown in Table 3. To exclude the effects of steroids on the results, we compared simple CSC and complex CSC patients who had not used steroids. The analysis revealed that in the absence of steroid use, the complex CSC group had significantly higher levels of VEGF, hs-CRP, and ESR compared to the simple CSC group (*P* < 0.05), as shown in Table 4.

### 3.4. Risk Factors for CSC

A logistic regression model was constructed with gender, smoking history, alcohol consumption, obstructive sleep apnea, hypothyroidism, kidney disease, *Helicobacter pylori* infection, corticosteroid use, shift work, VEGF, hs-CRP, and ESR as independent variables and CSC as the dependent variable. After backward elimination of nonsignificant variables (*P* > 0.05), male gender, shift work, *Helicobacter pylori* infection, hypothyroidism, elevated VEGF, hs-CRP, and ESR levels were identified as significant risk factors for CSC (*P* < 0.05), as shown in Table 5.

### 3.5. Diagnostic Value of VEGF, hs-CRP, and ESR in CSC

**Figure 2:**
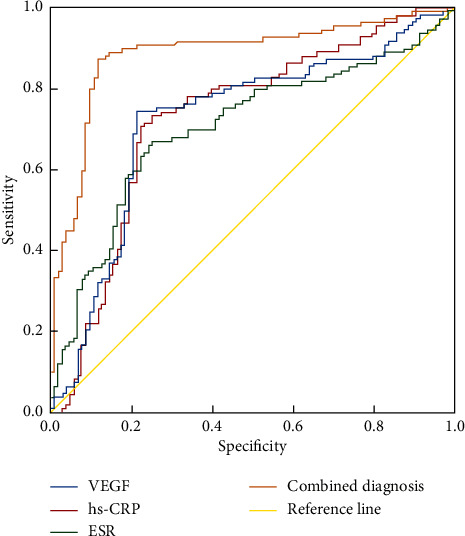
ROC diagram of VEGF, hs-CRP, and ESR in the diagnosis of CSC. Note: central serous chorioretinopathy (CSC), vascular endothelial growth factor (VEGF), high-sensitivity C-reactive protein (hs-CRP), erythrocyte sedimentation rate (ESR).

ROC curve analysis revealed that the combination of VEGF, hs-CRP, and ESR for diagnosing CSC yielded a significantly higher area under the curve (AUC) of 0.886 compared to using any single marker alone (VEGF: 0.722, hs-CRP: 0.728, ESR: 0.703; *P* < 0.05). This indicates that the combined use of these biomarkers improves the diagnostic efficiency of CSC, providing a novel approach for its diagnosis, as shown in Table 6 and Figure 2.

## 4. Discussion

CSC has emerged as the fourth most common retinal disorder following age-related macular degeneration, diabetic retinopathy, and retinal vein occlusion [21, 22]. Unlike other retinal diseases, CSC often resolves spontaneously without intervention, but some patients may experience visual impairment. Current treatment modalities for CSC include photodynamic therapy, oral mineralocorticoid antagonists, focal laser photocoagulation, and antivascular endothelial growth factor (VEGF) therapy [23, 24]. Despite their efficacy in improving visual outcomes, the recurrence rate remains high after cessation of treatment. Understanding the risk factors for CSC and analyzing relevant biological markers play crucial roles in clinical prevention strategies.

Our study identified male gender, shift work, *Helicobacter pylori* infection, and hypothyroidism as risk factors for CSC. The proportion of males in the CSC group was significantly higher compared to the control group, consistent with previous reports [21, 25]. Shift work, characterized by disruption of circadian rhythm, affects the hypothalamic-pituitary-adrenal axis, leading to dysregulated cortisol and catecholamine secretion, which may contribute to CSC development [26]. *Helicobacter pylori* infection, a common pathogen in gastric and upper gastrointestinal infections, can induce low-grade inflammation, endothelial cell damage in small arteries, focal choroidal capillary occlusion, ischemic changes, and upregulation of vascular endothelial growth factor expression, ultimately leading to choroidal neovascularization and leakage, hallmark features of CSC [27–29]. In our study, the prevalence of hypothyroidism was higher in the CSC group compared to the control group, consistent with previous findings indicating elevated serum thyroid-stimulating hormone levels in CSC patients, suggesting an association between hypothyroidism and CSC pathogenesis.

VEGF plays a pivotal role in angiogenesis and vascular permeability, promoting endothelial cell proliferation and neovascularization. Overexpression of VEGF has been implicated in various retinal diseases, including CSC, where anti-VEGF therapy has shown significant efficacy in improving visual outcomes [30]. Inflammatory cytokines exert profound effects on choroidal vascular permeability, and increased levels have been observed in the peripheral circulation of CSC patients. Inflammatory responses may induce elevated adrenaline levels, leading to choroidal vasoconstriction, microcirculatory disturbances, increased choroidal permeability, and subsequent CSC development [31]. High-sensitivity C-reactive protein (hs-CRP), a pentameric protein of the pentraxin family, synthesized by the liver in response to inflammatory stimuli, has been associated with exudative age-related macular degeneration. Erythrocyte sedimentation rate (ESR), a commonly used hematological marker, reflects increased inflammatory activity associated with autoimmune diseases, infections, or malignancies [32]. Our study demonstrated elevated levels of VEGF, hs-CRP, and ESR in the CSC group compared to the control group. Regression analysis identified elevated levels of VEGF, hs-CRP, and ESR as risk factors for CSC, suggesting that increased VEGF levels promote choroidal neovascularization and increased permeability, while elevated hs-CRP and ESR levels indicate enhanced systemic inflammation, exacerbating choroidal vascular dysfunction and microcirculatory impairment, leading to CSC development. ROC analysis indicated that VEGF, hs-CRP, and ESR have diagnostic value in CSC, with significantly improved diagnostic efficiency when used in combination, highlighting their potential as biological markers for CSC diagnosis. However, when using VEGF as a diagnostic biomarker for CSC, it is important to consider the disease form and duration. As a study shown that while some CSC patients have downregulated levels of vascular endothelial growth factor (VEGF), those with CSC complicated by choroidal neovascularization (CNV) exhibit significantly elevated levels of VEGF [14].

This study has several limitations that should be considered. Firstly, the sample size was relatively small, potentially limiting the generalizability of the findings. Secondly, the retrospective design introduces the possibility of recall bias and incomplete data. Additionally, while efforts were made to control for confounding variables, residual confounding may still exist. Moreover, the study's single-center nature may limit its external validity. Finally, the observational design precludes causal inference. Larger prospective studies are needed to validate these findings and elucidate causal relationships.

In summary, our study identified male gender, shift work, *Helicobacter pylori* infection, and hypothyroidism as predominant risk factors for CSC, with elevated levels of VEGF, hs-CRP, and ESR associated with CSC development. VEGF, hs-CRP, and ESR hold promise as biological markers for CSC diagnosis.

## Figures and Tables

**Table 1:** Comparison of baseline data between CSC group and control group.

Variables	CSC group (*n* = 109)	Control group (*n* = 103)	*t*/*χ*^2^ value	*P* value
Age (years)	40.12 ± 6.23	39.05 ± 6.41	1.232	0.219
Male/Female [case (%)]	80 (73.39)/29 (26.61)	51 (49.51)/52 (50.49)	12.791	0.000
Smoking history [case (%)]	55 (50.46)	32 (31.07)	8.229	0.004
Drinking history [case (%)]	59 (54.13)	39 (37.86)	5.636	0.018
Basic disease [case (%)]				
Hypertension	25 (22.94)	22 (21.36)	0.276	0.782
Diabetes	19 (17.43)	15 (14.56)	0.076	0.782
Hyperlipidemia	20 (18.35)	17 (16.50)	0.324	0.57
Comorbidity [case (%)]				
Obstructive sleep apnea	19 (17.43)	6 (5.83)	6.858	0.009
Hypothyroidism	21 (19.27)	5 (4.85)	10.222	0.001
Kidney disease	11 (10.09)	2 (1.94)	6.111	0.013
*Helicobacter Pylori* infection	45 (41.28)	20 (19.42)	11.911	0.001
Steroid usage history [case (%)]	25 (22.94)	7 (6.80)	10.764	0.001
Shift work [case (%)]	52 (47.71)	21 (20.39)	17.505	0.000
Antidepressant usage [case (%)]	13 (11.93)	9 (8.74)	0.579	0.447

*Note.* Central serous chorioretinopathy (CSC).

**Table 2:** Comparison of baseline data between simple CSC group and complex CSC group.

Variables	Simple CSC group (*n* = 57)	Complex CSC group (*n* = 52)	*t*/*χ*^2^ value	*P* value
Age (years)	41.55 ± 5.01	40.24 ± 6.21	−1.217	0.226
Male/Female [case (%)]	42 (73.68)/15 (26.32)	38 (73.08)/14 (26.92	0.005	0.944
Smoking history [case (%)]	29 (50.88)	26 (50.00)	0.008	0.929
Drinking history [case (%)]	31 (54.39)	28 (53.85)	0.003	0.956
Basic disease [case (%)]				
Hypertension	13 (22.81)	12 (23.08)	0.001	0.975
Diabetes	10 (17.54)	9 (17.31)	0.001	0.975
Hyperlipidemia	10 (17.54)	10 (19.23)	0.052	0.820
Comorbidity [case (%)]				
Obstructive sleep apnea	10 (17.54)	9 (17.31)	0.001	0.975
Hypothyroidism	11 (19.30)	8 (15.38)	0.289	0.591
Kidney disease	6 (10.53)	5 (9.62)	0.025	0.874
*Helicobacter Pylori* infection	24 (41.11)	21 (40.38)	0.033	0.856
Steroid usage history [case (%)]	13 (22.81)	12 (23.08)	0.001	0.975
Shift work [case (%)]	27 (47.37)	25 (53.85)	0.005	0.944
Antidepressant usage [case (%)]	7 (12.28)	6 (11.54)	0.014	0.906

*Note.* Central serous chorioretinopathy (CSC).

**Table 3:** Differences of VEGF, hs-CRP, and ESR between simple CSC group and complex CSC group.

Variables	Simple CSC group (*n* = 57)	Complex CSC group (*n* = 52)	*t*/*χ*^2^ value	*P* value
VEGF (pg/mL)	105.24 ± 23.41	128.56 ± 24.54	5.072	<0.001
hs-CRP (mg/L)	4.56 ± 1.13	5.72 ± 1.51	4.566	<0.001
ESR (mm/h)	18.15 ± 2.19	28.02 ± 2.05	24.227	<0.001

*Note.* Central serous chorioretinopathy (CSC), vascular endothelial growth factor (VEGF), high-sensitivity C-reactive protein (hs-CRP), erythrocyte sedimentation rate (ESR).

**Table 4:** Differences of VEGF, hs-CRP, and ESR between simple CSC and complex CSC without steroid use ($\overset{¯}{x}\pm s$).

Groups	n	VEGF (pg/mL)	hs-CRP (mg/L)	ESR (mm/h)
No steroids simple CSC group	10	112.24 ± 17.97	4.85 ± 0.89	22.02 ± 2.07
No steroids complex CSC group	74	123.72 ± 18.12	5.53 ± 0.91	24.07 ± 2.23
*t*		2.086	2.223	2.749
*P*		0.040	0.029	0.007

**Table 5:** Logistic regression model of risk factors for CSC.

Factor	*β*	Wald *χ*^2^	OR (95% CI)	*P* value
Male	0.823	16.436	2.277 (1.530∼3.390)	0.000
Night shift work	0.729	13.976	2.073 (1.415∼3.038)	0.000
*Helicobacter pylori* infection	0.632	11.671	1.881 (1.309∼2.704)	0.001
Hypothyroidism	0.559	18.209	1.749 (1.353∼3.261)	0.000
VEGF	0.495	10.332	1.640 (1.213∼2.219)	0.000
hs-CRP	0.632	16.204	1.881 (1.383∼2.559)	0.000
ESR	0.495	13.247	1.640 (1.257∼2.142)	0.000

*Note.* Central serous chorioretinopathy (CSC), vascular endothelial growth factor (VEGF), high-sensitivity C-reactive protein (hs-CRP), erythrocyte sedimentation rate (ESR).

**Table 6:** Efficacy of VEGF, hs-CRP and ESR in the diagnosis of CSC.

Factor	Area under curve (95% CI)	Threshold	Sensitivity (%)	Specificity (%)	Youden's index
VEGF	0.722 (0.650∼0.793)	89.15 pg/mL	75.23	73.79	0.4902
hs-CRP	0.728 (0.658∼0.799)	3.14 mg/L	70.64	77.67	0.483
ESR	0.703 (0.631∼0.775)	19.05 mm/h	66.97	72.82	0.3979
Combined	0.886 (0.837∼0.934)	—	87.16	88.35	0.7551

*Note.* Central serous chorioretinopathy (CSC), vascular endothelial growth factor (VEGF), high-sensitivity C-reactive protein (hs-CRP), erythrocyte sedimentation rate (ESR).

## Data Availability

The datasets generated and analyzed during the current study are available from the corresponding author on reasonable request.
